# Application of CRISPR/Cas9 technology in wild apple (*Malus sieverii*) for paired sites gene editing

**DOI:** 10.1186/s13007-021-00769-8

**Published:** 2021-07-19

**Authors:** Yan Zhang, Ping Zhou, Tohir A. Bozorov, Daoyuan Zhang

**Affiliations:** 1 State Key Laboratory of Desert and Oasis Ecology, Xinjiang Institute of Ecology and Geography, Chinese Academy of Sciences, Urumqi, 830011 China; 2 Xinjiang Key Laboratory of Stress Resistant Plant Conservation and Research, Xinjiang Institute of Ecology and Geography, Chinese Academy of Sciences, Urumqi, 830011 China; 3 University of Chinese Academy of Sciences, Beijing, 100049 China

**Keywords:** CRISPR/Cas9, Gene editing, *Malus sieverii*, *PDS* gene

## Abstract

**Background:**

Xinjiang wild apple is an important tree of the Tianshan Mountains, and in recent years, it has undergone destruction by many biotic and abiotic stress and human activities. It is necessary to use new technologies to research its genomic function and molecular improvement. The clustered regulatory interspaced short palindromic repeats (CRISPR)/CRISPR-associated protein (Cas) system has been successfully applied to genetic improvement in many crops, but its editing capability varies depending on the different combinations of the synthetic guide RNA (sgRNA) and Cas9 protein expression devices.

**Results:**

In this study, we used 2 systems of vectors with paired sgRNAs targeting to *MsPDS*. As expected, we successfully induced the albino phenotype of calli and buds in both systems.

**Conclusions:**

We conclude that CRISPR/Cas9 is a powerful system for editing the wild apple genome and expands the range of plants available for gene editing.

**Supplementary Information:**

The online version contains supplementary material available at 10.1186/s13007-021-00769-8.

## Background

Wild apple (*Malus sieversii* (Ledeb*.*) *Roem.)*, is native to the mountains of Central Asia and is widely distributed in the wild fruit forests of the Tianshan Mountains. Wild apple forests are estimated to account for 92% of the fruit forests of the Chinese Tianshan Mountains, whereas wild apple forests make up approximately 78% of the wild apple forests in the Central Asian Tianshan Mountains [1]. Evolutionarily, the wild apple population has evolved cold-tolerant and disease-resistant varieties with diverse fruit colourations, shapes, and flavours [1]. Many genetic studies have shown that the wild apple *M. sieversii* is the distant ancestor species of cultivated domesticated apple species [2–4]. Unfortunately, in the last two decades, wild apple trees in Tianshan forests have been heavily damaged by biotic stresses and human activities that have resulted in severe forest destruction [5–7]. This tree has been listed as an endangered second-class protected plant in China [8]. Therefore, this valuable germplasm urgently needs effective protection, and it is necessary to create new stress-resistant germplasms. Due to the self-incompatibility of wild apples and their longer time to maturity [9], conventional breeding methods cannot easily meet the urgent needs.

Genome editing tools include zinc-finger nucleases (zinc-fingernuclease), transcription activator-like effector nuclease (TALEN), and the clustered regulatory interspaced short palindromic repeats (CRISPR)/CRISPR-associated protein (Cas) system (CRISPR/Cas) [10]. Compared with the other techniques, CRISPR/Cas9 has become a powerful technology for gene editing because of its easy construction and high specific efficiency. The CRISPR/Cas system can be divided into six types (types I–VI) [11]. Type II Cas9 protein has been widely adopted as a simple and highly efficient targeted genome editing tool for multiplex genome engineering using CRISPR/Cas systems [12]. In this system, the mature dual CRISPR RNA consists of a crRNA and small trans-activating CRISPR RNA (tracrRNA), which forms a functional complex with the endonuclease Cas9. Scientists combined the two small RNAs into a single guide RNA (sgRNA), which was more convenient to use. The Cas9 ribonucleoprotein complex then binds to DNA at a typical protospacer adjacent motif (PAM) sequence and a protospacer matching the crRNA through Watson–Crick pairing [13, 14]. Cleavage occurs at the targeted site concerning the PAM on both strands, mediated by the Cas9 endonuclease domains RuvC and HNH, introducing precise double-strand breaks (DSBs) [13]. DSBs can be repaired in two ways, nonhomologous end joining (NEHJ) and homology-directed repair (HDR), depending on the cell type, target site, and DNA repair machinery [15]. NHEJ is responsible for the vast majority of repair and may occur in almost all types of cells as well as in different phases of the cell cycle (G1, S, and G2 phases), introducing various indels and substitutions at DSB sites that cause gene knockout or knockdown [16, 17]. HDR events occur mainly during the S and G2 periods if a homologous DNA donor is available to serve as a repair template [18, 19]. HDR-based repair has the greatest potential for precise genome editing but currently suffers from very low efficiencies. For eukaryotic genome editing, nuclear localization signals (NLSs) are added to the Cas9 protein to encourage nuclear localization [12, 20]. The Cas9 gene is driven by a constituent promoter with high transcriptional activity, such as the Cauliflower mosaic virus (CaMV) 35S promoter or the Arabidopsis ubiquitin 10 (*AtUbi*) promoter, to drive Cas9 expression in dicots, Maize or rice ubiquitin (*ZmUbi*; *OsUbi*) promoters in monocots [15]. Some organ-specific promoters are also used to drive Cas9 expression, such as the female gametophyte specificity promoter [21], male gametophyte-specific promoter [22], meristem promoter [23], and *yao* promoter [24]. sgRNAs are typically expressed under small nuclear RNA promoters, such as the U6 and U3 promoters constitutively transcribed by RNA polymerase III (Pol III) [25–29]. To edit multiple genes simultaneously, multiple sgRNA expression cassettes harbouring a small RNA promoter (U6 or U3), sgRNA spacer targeting sequence, sgRNA scaffold sequence, and 3’ terminator element were combined in series by the Golden Gate cloning method [29–31]. In planta expression of multiplexed gRNAs can also be based on ribozyme processing, Csy4 processing, or tRNA processing [15]. A polycistronic tRNA–sgRNA system generates multiple sgRNAs by using tRNA precursor sequences (pre-RNA) to link the sgRNA sequences under transcription by a U3 or U6 promoter [32]. CRISPR/Cas9 technology has been applied to wheat, rice, citrus, lettuce, and other fruits, and some new germplasms have been created, such as anti-powdery mildew wheat, ulcer-resistant citrus, and herbicide-resistant rice [33–38]. Phytoene desaturase (*PDS*) gene is a key gene in chlorophyll synthesis, and its mutant plants with albino phenotype and has served as a model gene for CRISPR/Cas9-mediated targeted gene editing [34, 37, 39]. In this study, we used two different systems to construct 8 different vectors with pairing sgRNAs targeting the *MsPDS* gene. Some calli and buds with mutant were successfully obtained. This is the first successful application of the CRISPR/Cas9 gene editing system in this species, which will provide strong technical support for its gene function research, utilization, and conservation.

## Results

### Cloning of *MsPDS* and selection of target sites

**Fig. 1:**
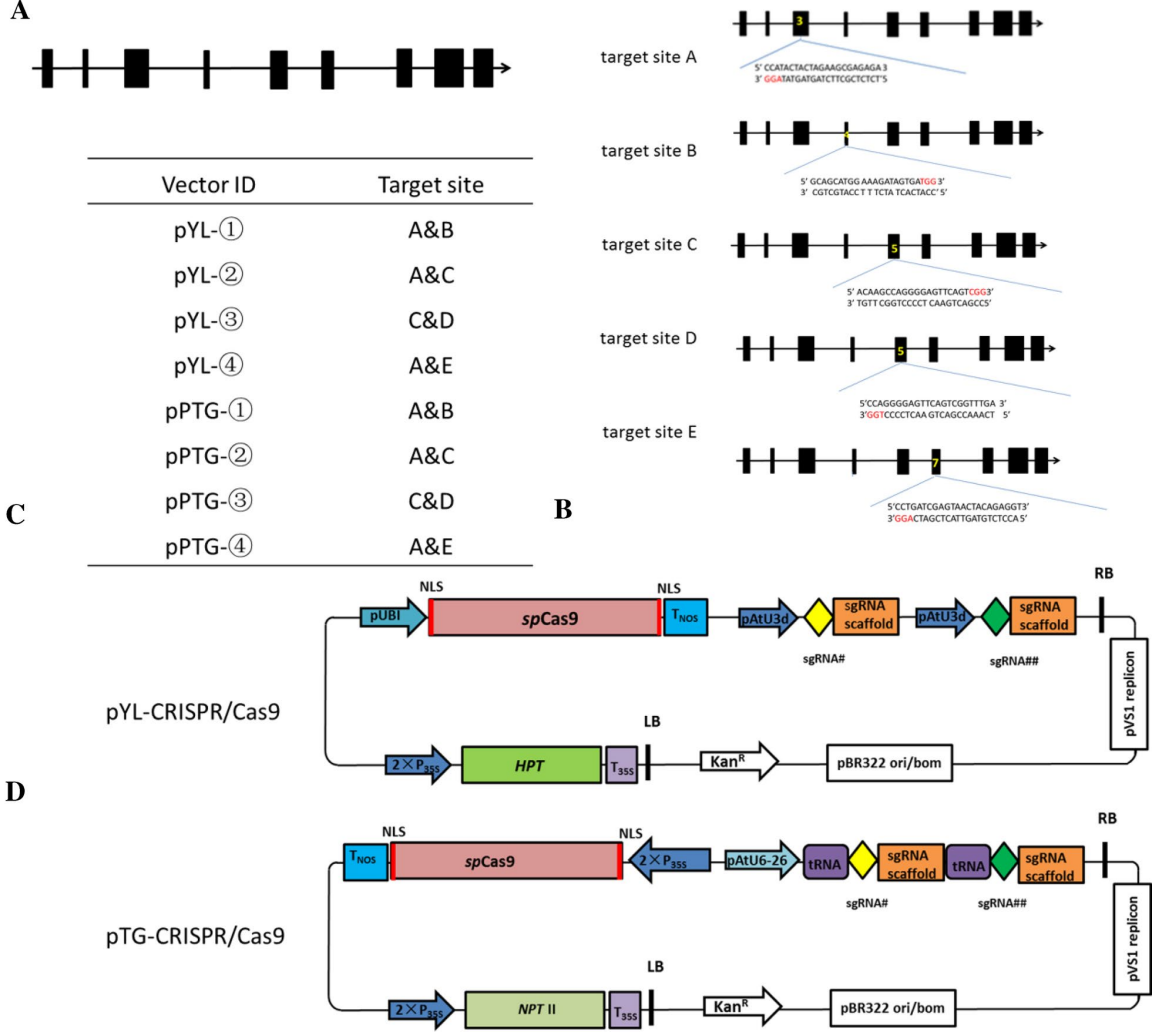
The structure of *the MsPDS* gene, target site selection, and schematic diagram of the Cas9/sgRNA vector. **A** The structure of *the MsPDS* gene. The black rectangles represent the different exons. **B** The target sites of different exons. The yellow number is the position of the exon. The red text indicates the PAM. **C** The vector ID of paired-sgRNA/Cas9 binary vectors for both the CRISPR/Cas9 and pTG-CRISPR/Cas9 systems. **D** Schematic depicting the paired sgRNA expression cassettes of both the CRISPR/Cas9 and pTG-CRISPR/Cas9 systems. Diamonds of different colours represent sgRNA, rectangles represent the sgRNA scaffold, and purple rounded rectangles represent tRNA

Two pairs of primers were used to clone the approximately full-length *MsPDS* gene sequence 5232 bp (supplementary sequence). The intron and exon sequences of this gene have been determined by the sequence alignment of multiple species. Five target sites (Fig. 1B) in exons 3, 4, 5, and 7 were selected and termed as target sites A, B, C, D, and E, respectively. According to the target site sequence, a 20 bp sgRNA was designed.

### Constructs for the targeted genome editing of wild apple

To edit the target gene, we selected two different systems, pYL-CRISPR/Cas9 and pTG-CRISPR/Cas9, and constructed 8 different plasmids harbouring 2 sgRNAs according to their methods (Fig. 1C) [33, 40, 41]. For the pYL system, Cas9 endonuclease was under the maize ubiquitin promoter (P_UBI_), and a sgRNA expression cassette was under the *AtU3d* promoter. This system used the hygromycin resistance cassette in the vector for the selection of wild apple transformants. In contrast, for the pTG-CRISPR/Cas9 system, the same Cas9 endonuclease was located under the cauliflower mosaic virus 35S promoter (P_35S_) promoter, and 2 sgRNA expression cassettes were located under the *AtU6-26* promoter and blanked by tRNA^Gly^, which may function as a transcriptional enhancer for pol III promoters. This system used the *NPT* II resistance cassette in the vector for the selection of wild apple transformants. All 8 constructs with different paired sgRNAs (Fig. 1C) were transformed into wild apple leaf discs separately using the *Agrobacterium* method.

### Efficiency of calli editing

**Fig. 2:**
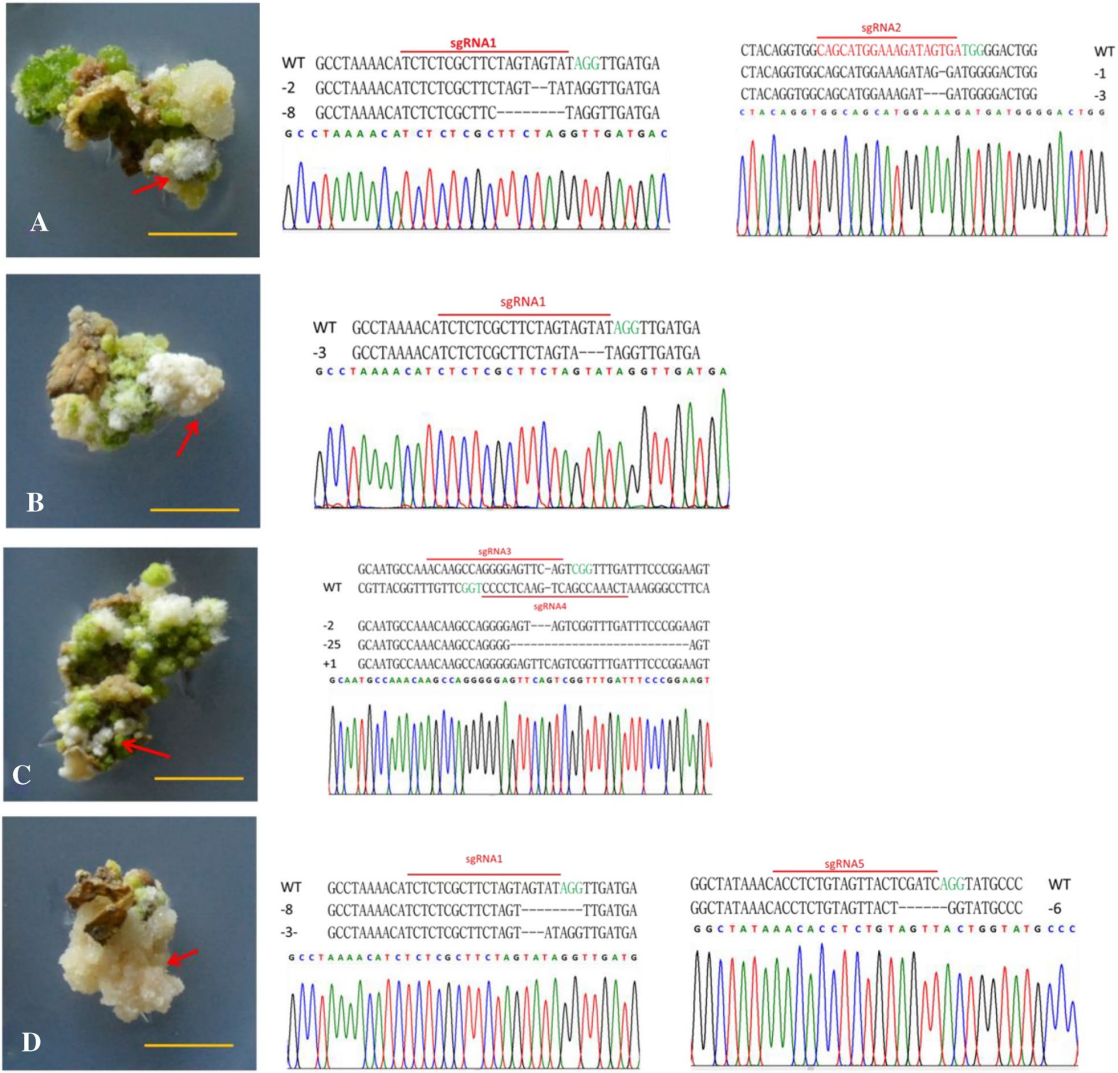
Mutant calli and Some Sanger sequencing of the target site. **A**–**D** left: Calli transformed by pYL-①, pYL-②, C: pTG-③, and pTG-④. Right: mainly mutation types and one sequencing chromatogram among them. Red arrow: some white calli in which *MsPDS* was edited

After 4 months of screening, some 1 cm^2^ resistant calli were obtained (Fig. 2). Typically, most of the wild-type calli were green after exposure to light. As expected, there were some white calli in the screening medium (Fig. 2). The calli were selected randomly to identify the mutation. The specific primers SP-L1/SP-R-L or SP-DL/SP-R-w of the T-DNA region and the *Agrobacterium*-specific primers VCF/VCR were used in PCR amplification, which revealed that the T-DNA region was successfully and efficiently inserted into the wild apple genome by both the pYL-CRISPR/Cas9 and pTG-CRISPR/Cas9 systems. Most of the resistant calli selected were transgenic. A total of 87.5% of the calli from hygromycin plates had the Cas9 gene inserted successfully, and 96.2% of the calli from kanamycin plates had the Cas9 gene inserted successfully (Additional file 1: Fig. S1A). Further, we detected whether the mutation was produced in these T-DNA insertion samples. PCR was performed using site-specific primers, and the PCR products were denatured and reannealed for the subsequent T7 endonuclease I assay (T7E1) (Additional file 1: Fig. S1B–D). The mutation efficiency of different loci was different (20–80%) (Table 1). The pYL-CRISPR/Cas9 system performed better than the pTG-CRISPR/Cas9 system in wild apple genome editing. The average target efficiency of pYL-CRISPR/Cas9 was 70.2%, while that of the pTG-CRISPR/Cas9 system was 40.3%. The efficiency of the two targets simultaneous editing was 59.5% for pYL-CRISPR/Cas9 and 26.6% for pTG-CRISPR/Cas9. The efficiency of different targets was different: target C had the highest average mutation rate, at 70%, and the mutation rate of E targets was 31.5%. To precisely identify the mutations in the resistant callus lines, the PCR products were purified using a cycle pure kit gel and cloned into a pMD20T vector followed by Sanger sequencing for validation. Mutation types were diverse and included not only 1–28 bp deletions but also 1–2 bp inserts (Fig. 2, Additional file 5).

**Table 1:** Summary of site-specific mutagenesis frequencies in *M. sieverii* callus

Vector ID	Target site	Number of callus lines analysed	Number of mutated callus lines	Mutation frequency (%)
pYL-①	A	18	13	72.2
	B	18	10	55.6
	A&B	18	10	55.6
pYL-②	A	21	14	66.7
	C	21	19	90.0
	A&C	21	14	66.7
pYL-③	C&D	23	17	73.9
pYL-④	A	19	18	94.0
	E	19	8	42.0
	A&E	19	8	42.1
pTG-①	A	10	4	40.0
	B	10	0	0
	A&B	10	0	0
pTG-②	A	20	9	45.0
	C	20	10	50.0
	A&C	20	9	45.0
pTG-③	C&D	5	2	40
pTG-④	A	14	12	85.7
	E	14	3	21.4
	A&E	14	3	21.4

### Mutation in wild apple buds

**Fig. 3:**
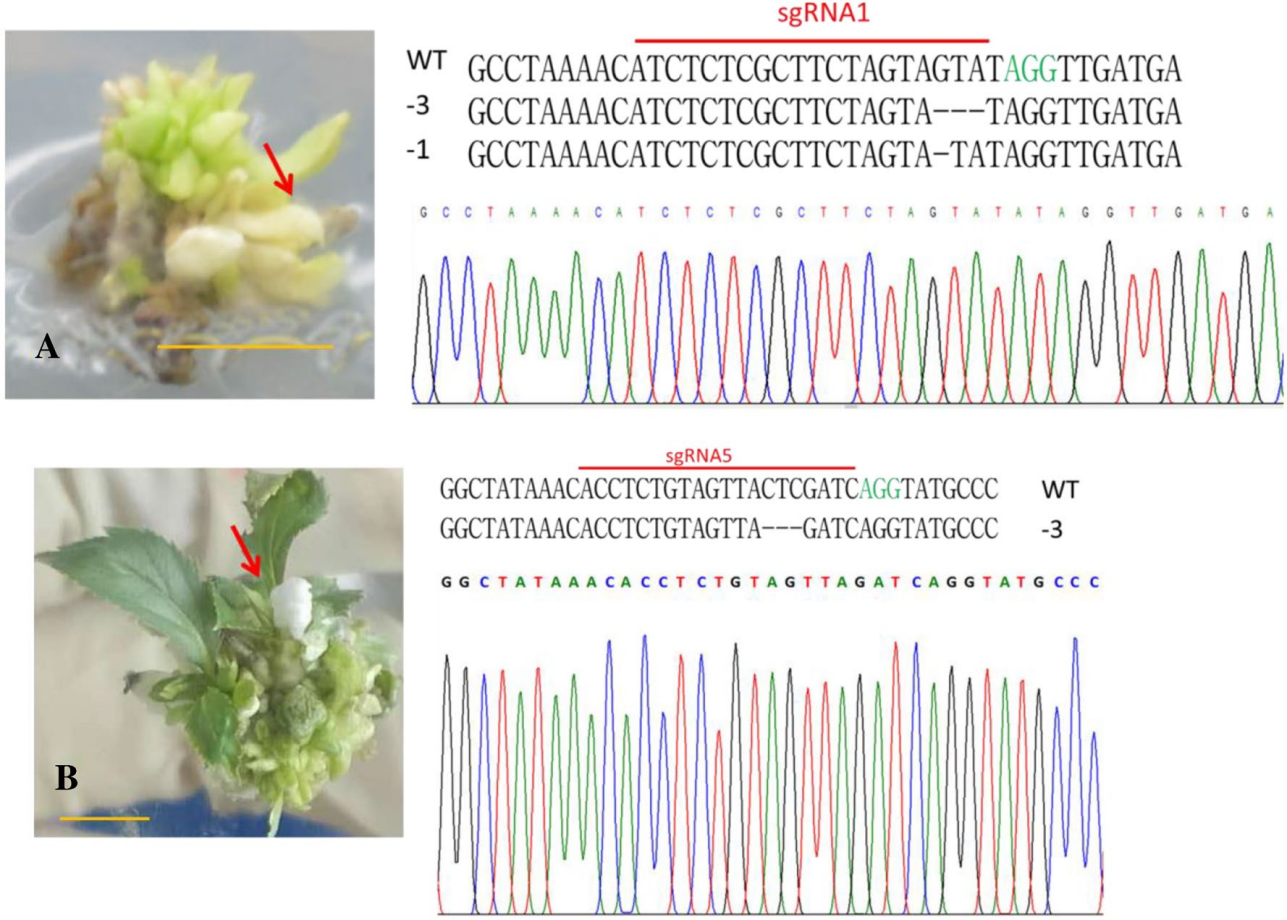
Mutant buds and Sanger sequencing of the target site. **A**, **B** left: Buds transformed by pYL-② and pTG-④, respectively. Right: mutation types and one sequencing chromatogram among them. Red arrow: albino parts in which *MsPDS* was edited

For the pYL system, we obtain 1 bud which *PDS* gene was edited through transformation method 1. Half of it is green, and the other is pale. Sanger sequencing shows there is 2 mutant type in the albino parts of bud (Fig. 3A), while one mutant is same as calli. For the pTG system, through method 2, we only obtained 1bud with one leaf albino (Fig. 3B). Because screening pressure was not applied in the cultured early stage, the bud is chimeric. Through Sanger sequencing, there is 3 base deletion in target 5 (Fig. 3B).

### Off-target probability

To date, it is difficult to evaluate the off-target results of gene editing because the whole genome of *M. sieversii* has not been published. We designed 5 different sgRNAs using an online website and evaluated their potential off-target rate (< 0.6, good) (Additional file 3) by comparison to the apple genome (GDDH13, Version 1.1). Eighteen of the 54 potential off-target sites are in the CDS region (Additional file 3). For a preliminary evaluation of the off-target, we cloned ~ 300 bp upstream and downstream of each of these genes from the *M. sieversii* genome (Additional file 3, Additional file 4). Comparing with apple, these sequences are also highly conserved in wild apple. Sanger sequencing revealed no mutation in these DNA samples(Additional file 4), which were randomly selected from samples with the *PDS* gene edited by CRISPR/Cas9. Preliminarily, it was proven that these two systems have high fidelity in *M. sieversii*. In the future, it will be necessary to sequence the genome of *M. sieversii* and comprehensively evaluate the off-target rate caused by gene editing.

### Discussion

The CRISPR/Cas9 system is a powerful genetic editing tool. To date, rice, corn, wheat, tomato, apple, grape, kiwifruit, and other food or cash crops have been edited [35, 42]. pYL-CRISPR/Cas9 is a multi-target system developed by Pro. Liu [36], and the pTG-CRISPR/Cas9 system is another paired targeting system designed by Dr. Huang that was used for kiwifruit efficiency editing [33]. Both systems were used for the genome editing of wild apple, and our results show that gene editing technology can be applied in the wild apple. Our data revealed consistently high editing efficacy in both systems, which is different from the previous results in kiwifruit. Perhaps the P_UBI_ is as efficient as the P_35S_ in wild apple. In our study, it was easy to obtain edited callus, while only a few buds with edited were obtained. The regeneration of plant becomes a limiting factor to apply this technology. Woody plant is hard to separate CRISPR/Cas9 by breeding in contrast to crops owing to it need long time to maturity. Some scientists used RNPs to edit genomic genes in apple protoplast [35]. On the other hand, some scientists have tried to use the loxp or FLP/FRT method to delete the Cas9 and sgRNA genes [43, 44]. But, there are some residual T-DNA sequences in a plant. T-DNA free is another limiting factor to apply this technology. Researching key genes concerning plant regeneration and T-DNA integration into the chromosome is the way to solve these problems.

In spite of this, it is easy to obtain edited calli, which is also the material for gene function study. Chen used apple calli to analyze the functions of *MdMYBPA1*and *MdSYP*121 in anthocyanin synthesis and the activation of disease resistance against *Botryosphaeria dothidea* [45, 46]*.* Using our experimental method, gene-edited calli can be obtained within 4 months. The establishment of this method contributes to the study of the genetic function of *Malus* plants.

## Conclusions

In summary, we have used the CRISPR/Cas system to edit the genome of Xinjiang wild apple and for the first time edited two targets at the same time. The success of this experiment expanded the range of plant species that can be edited by the CRISPR/Cas9 system and will provide technical support for the creation of new germplasms and research on functional genes in *M. sieversii*.

## Methods

### Cloning of the *PDS* gene of *M. sieversii*

Genomic DNA was extracted from wild-type plants using the cetyltrimethyl ammonium bromide (CTAB) method [47]. Two pairs of primers (Ms-PDS-F1/Ms-PDS-R1 and Ms-PDS-F2/Ms-PDS-R2) were used to clone this gene. To analyse the intron–exon structure, the sequence was compared with reported apple *PDS* ESTs (accession nos. GO517828.1, GO499218.1, GO523095.1, GO514137.1, GO547261.1) and *PDS* ESTs from* Arabidopsis* (L16237), tomato (X59948), pepper (X68058), and soybean (M64704) by the methods reported by Chikako et al. [34].

### pYL-CRISPR/Cas9 and pTG-CRISPR/Cas9 expression vector construction

pYL-CRISPR/Cas9 vector in which Cas9 is driven by the maize ubiquitin promoter(P_UBI_) contains hygromycin plant selectable marker gene and has two *Bsa*I sites that flank a toxic *ccdB* gene for cloning of sgRNA expression cassettes. pTG-CRISPR/Cas9 vector in which Cas9 is driven by the cauliflower mosaic virus 35S promoter (P_35S_) contains *NPT*II (kanamycin resistance) plant selectable marker gene. Five sgRNAs targeting the different exons of the *MsPDS* gene were designed, termed sgRNA1 to sgRNA5. Each pair of targets was cloned into a single Cas9 binary vector to construct a paired-sgRNA/Cas9 binary. For the pYL-CRISPR/Cas9 system, sgRNA was first introduced into the region downstream of the U3d promoter and upstream of the sgRNA scaffold by overlapping PCR through the intermediate vector pYL-sgRNA-AtU3d. Then, 2 different sgRNA expression cassettes were constructed in the pYL-CRISPR/Cas9 vector by *BsaI* digestion and T4 ligation Golden Gate cloning [41]. For the pTG-CRISPR/Cas9 system, 2 different sgRNAs were first introduced downstream of the tRNA and upstream of the sgRNA scaffold by overlapping PCR through the intermediate vector pHLW-gRNA-tRNA, and then the sgRNA expression cassette was cloned into the pTG-CRISPR/Cas9 vector by *BsaI* digestion and T4 ligation Golden Gate Cloning [33]. The sgRNA oligos are shown in Additional file 2.

### Tissue culture and *Agrobacterium*-mediated transformation

*M. sieversii* tissue culture was performed as previously described [48], and the leaf discs were used for *Agrobacterium* (EHA105)-mediated transformation. To obtain a stable transformation bud, we adopted two different genetic transformation methods owing to the extremely low genetic transformation efficiency. In the first method, after the leaf is infected by *Agrobacterium*, it grows on the screening medium, and a large number of resistant calli can be obtained after four months. The shortcoming of this method is obtaining fewer transformation buds. In the second method, the leaf discs are infected by *Agrobacterium* cultured in the bacteriostatic medium and given time for the adventitious buds to grow, and then the buds are transferred to the screening medium.

### Mutant detection

Genomic DNA was extracted from resistant callus lines and regenerated buds. To validate the T-DNA insertion, *Agrobacterium*-specific primers (VCF/VCR) [49] and SP-L1/SP-R-L or SP-DL/SP-R-W of the T-DNA region were used. The mutation efficiencies were examined as the ratio of callus lines with a mutation. The fragments encompassing each target were separately amplified using the target-specific primers (Additional file 2). Approximately 500 ng of the respective PCR product was used for the T7E1 array with T7 endonuclease I according to the manufacturer’s instructions. After the T7E1 array, the reaction products were analysed by 1.5% agarose gel electrophoresis. Besides, to find out the specific type of mutant, the PCR products were cloned into the pMD20T vector. The ligated products were transformed into *Escherichia coli* strain DH5a cells, and clones were selected for Sanger sequencing.

### Off-target analysis

The potential off target sites was predicted by reference *Malus domestica* genome(GDDH13, Version1.1) using the online tool CRISPR-GE (http://skl.scau.edu.cn/), and 9 potential off-target sites were retained. Site special primers were designed to clone these sequences in *M.sieverii*. 20 to 40 DNA with *PDS* edited were used for off-target analysis. PCR products 10 in a group were sequencing by Sanger sequence using sequencing primer (Additional file 3).

## Supplementary Information

**Additional file 1.** *MsPDS* sequence, Supplement Table 1 Selection of target sites and off target possibility, Supplementary Fig 1 Identification of T-DNA insertion and indel detection by T7E1 assay.

**Additional file 2.** Primer used in this study.

**Additional file 3.** Predicted Off-target site and related primers.

**Additional file 4.** Off target site Sanger sequencing.

**Additional file 5.** Sanger sequencing of target site.

## Data Availability

The datasets used and/or analysed during the current study are available from the corresponding author on reasonable request.
