# Supplementary material for: Application of CRISPR/Cas9 technology in wild apple (Malus sieverii) for paired sites gene editing
Source: Plant Methods. 2021 Jul 19;17:79. doi: 10.1186/s13007-021-00769-8 (PMC8287690; doi:10.1186/s13007-021-00769-8)
Supplement: Supplementary file 1 — Additional file 1. MsPDS sequence, Supplement Table 1 Selection of target sites and off target possibility, Supplementary Fig 1 Identification of T-DNA insertion and indel detection by T7E1 assay. [file 13007_2021_769_MOESM1_ESM.docx]

# Additional Materials for

Application of CRISPR/Cas9 Technology in Wild Apple (Malus *sieverii*) for gene editing

MsPDS sequence

>MsPDS

TTCATCACTCATCGCACTCGCACACCTTTCCTCCTCTCCCTCACCCAAATTTGGAGCTAAAAAAAGCTCCATTTCTTGGCGGGGGCTGAGACTCTTGATCTGCGCGCAACCTGTTTGATAAAATGGCGCAGTGGGCTTGTGTCTCCGCTGCTAACTTGAGCTGCCAAGCTACCATCGTAAACACTCAAAAGCAACGAAACAGTCCCGGATGCGATGCCTTTTCTTTCAAAGGCAGTGAATTTATGGCTCAGAGCTGTAGATTTTCAAGCCCACAAGCTGTTTATAGAAGGCCCAGGAATGGTGTTTGCCCCTTGAAGGTTTGTTTTCTGTAATTTTTTTAGTGTTTTTGTTGCTAAATTGTTGAATTGTTAAAAGGGTGGTCAAGTTATATGTGTGTGATTGTAATGGCATAGGTGGTTTGCGTTGATTATCCAAGACCAGACCTTGACAGTACTGCTAATTTCTTAGAAGCTGCGTACTTCTCTTCCACTTTCCGAGCCTCTCCTCGTCCAACCAAGCCGTTAAAAGTTGTGATTGCTGGTGCAGGTGATTAATTCAATCTGATTTTATATTTTTTGGAAAATCAATTCTTAAATTGCTTAGAACTGAATTGGGTTTTATTCTTCTGTTTATAATTTGTCTCATAGGAATCCTGTATGTATACGAAATATAGCATTTTCTCTTTTATTAAGGGATTAATATTTATTTTCTATATGTTTTTGCAGGTTTGGCTGGTCTGGCAACTGCAAAATATTTGGCGGATGCGGGTCATCAACCTATACTACTAGAAGCGAGAGATGTTTTAGGCGGAAAGGTTTTCGGCCGCTCTAACTTTATCCTTCTATGAAACCATATTGCCGTTAGCTTGAAAATGTTATAACTTACATGTTTCATAGCTAATAATTCGGAATCTCTTTTAAAATTTACCAAAGGAAACATAAGGAACAGAGACATAATCCATTAGTAGGAGAGCAAGGGAAGGATTGTTTTACATGCTTAAGGAAATTTATTATGAGTTGCAAAAATGAAACACACTAATTTCTTGAAACCTATTGCCATCTAAAGACAAAGTGCAAAAGTAGAAGCTTCAGTCTTACATTATGAAGCTATATGTTATCTTTGGTTACGATACAATTTGCTCCTCCATGGAATGAAACTGGTGGAATTTGGGGACCTCGTATAGTTGTGCTTTCTAGTCTTTCGACTTATGTCTTGTCCTTGTTTTTATCTCAAACCATTGCCCAATAGTTTAGTCAAGCAAGACAGAATGGATTTTCATTTTAGCATGTTTGAATGCTGAGTTTCAGTACCATTATTGATTAGGACATATTTTCTTATGATGAAGGATGGTGGGTTAGGAATTATTGAGTTGAGTGCTTTTGATCAGACACTCTTATCTGTTGGTGGCGGGGTGTGTGTTGGGATGGGGGATGTGTTGTCTGACAGGTCCTAAATGTGTAAGGGTGCCCTGCTATTTATTTAGGTGCTAACAGTAGCAGCCATTTAGAGGTCATTGTCCAATTATATCAACATTCTAACTCGATAGGTCTGAAATCGTTTTTTTTTTCTTATTTAACTCTCCATTTTTTCTACAGGTGGCAGCATGGAAAGATAGTGATGGGGACTGGTATGAAACAGGCCTGCATATATTCTGTGAGTTTAATTTTTCTCATTTACTTATACGAAAATGCTTTTTTAAACTTATAGAGAGGTCCTTTTTCCAAATAGATTCTTCATGCTACATTTGGGTGGCTGGTATAGGAGAGGATGATGGAGTGTAACAGTGTAACAAATCTGGGAATGAGATTCCTAGTAGTTAGTAGCTTGCATTATTCTCCTTCCAGTGTTTGGTTCAAAGTTCAGACTGTGCCAGGGTACATGGAGTCCACTATTTATGTTTTATATGTAGAAAATAAAATAAAATTCCTCTTTAGAGGTATTGAATGAGAGCAAATTACACTGTCAGGTAATTACGCTGGCAAGTGATTATCTTCTCTGAAAAGATAAAATGAAAGTCAGATTGAGATGTTCACGAAGCTGAGGAGAAGAATTTGCCGATGTATAAATTTATCATATTATATATCACTACAATATTTTAGCTAACAAGCAAGCTGCAACAGAGGGAGGAATAAAACTAATTCTGGGTGAACGTTGCTATATGTTAATGAAACAAAATGTTTAGGATTATATTGATTAGGAATGTTCTCTTGGTCATGAGGAGACTAAGAAATGTATGGTCACTCGCCCTTGGATTTGATATGAAGGGTGGAAAATAAATGGATGTGCTTATCTTTTAATCTCAACCCTTGATATCAAATTTAAGGGAATCCTTGGTCACCTGGTGACCAAGAGAACGGGACACTATATTTGATGGGGTTTTGAAACAGTACTTACAAAATCAGAAAATTTAAAGGTAGCTGTACCTAACATCTTAGGGGTTCGAACCCCAGGATTTCCAGCATCTGTTTTCATTGTCATCTTCTGTCCAAGGTGGATCTACATTATTTACACATTGCTTAATGAAACTGGTTGTATTTCTTTGCTTTAGGAATGTTCTATAAAACCAGATTACTGTCGTCTAGTGCTTTTACATAGACAGTTGCACAGTGGATTTAATTTCTTATGATGTAATTCTTTTTGTTTGTGTTGGCAAGTTGGGGCATATCCAAATATTCAGAATCTGTTTGGAGAGCTTGGTATTAATGATCGGTTGCAGTGGAAGGAACATTCTATGATATTTGCAATGCCAAACAAGCCAGGGGAGTTCAGTCGGTTTGATTTCCCGGAAGTTCTGCCAGCACCCATAAATGGTAAATATTACAGTCACCTCTTGAAGATTGAAAGTAGTTTGTTTACAAATGCAGAAAATTCTTTCTGTTCCTGGGAAGGTACCTTGCTTTATACACGCATTACAAAATACTTCATAGATGGAAGTACTGCAATACCTAGTCTATCCAATTGTCTGTCTATATTTGATAACCAACGCTCGCACTGCCAACCCTTTATTATCAACTGTGACCTAAAGACCAAAACATGAATGAAAGAACAACAATTCCTCATTGTAGCTTTAGGGCACCCTGCAAATATTCATACAAATATGTCTTTACTCGCATTGTAAAAGACAATTTTAAAAGTTCGATTCAAATTCTGGGAGACAACCCTGTTGCTTCATGATCAGATGATATACAGCTGTGTTCCATGTCACTGTGTATTGTTACTTTTTTGAGCTTCTTGTGATTGCGGCTTGCAGGAATATGGGCCATATTGAAGAACAATGAGATGCTGACTTGGCCAGAGAAAATCAAGTTTGCAATTGGACTACTGCCAGCAATCCTTGGTGGGCAGGCTTATGTTGAAGCCCAAGATGGCTTGAGCGTAAAAGACTGGATGAGGAAACAGGTACTCTAAAATATTTGATCATGTTCTTTTTGCATGTTTTATTGAGCGAGTCACGTGTTGTGACATATTATTAGGCTAGCTTATGTGCCATGAACACATCTCATAATGAGTTGAAAGTTAAAGAGTTAACATAAGAGGATTGTGCTTAGACAAACCTGCTTCCTGATCCTTCAGTTTCACAACATGAACTGTGCCAAGGTTGGTTGATCTCAGTACAGTATGCTGAGAATATTAATCTAGTCAACATTCCTGGTACTAGCAGATAGACAATGAAATTATTAAAATGAGATTTGCTGTACTTGTAAATTATTAGTCTATAATAAGACACCACTTATTATGGCACATAGAAATCCTATTTGGCACTAGTTTGGATTGTGAAATTCTGTAGGCACCAGTATCTGGTTTTCCATTTACTGCTTTTGTTTCATAATGGTCAAGATGTGAATGAGGCAGTGCTAGCCTCGTGAATTTTGAGAAGAAGAGGTAGGAGTTTTGAAAAAATTATCGAAATCTTAGGAAAAAAAACCTAAGAAAATTGAACTGTTTTCCTCTTTGAAGGAAAAATAGGCATAAGCTTATACATGCTCATAATTAATTATAACTGAAATCCACAAAATTACATGTCCAGTGAGAGAACAAGAGATACTTACAGAAATAAAAAAAAAACTGAGAGATTGCGACAGAATACTATGCTTGTCTTAGTGATTTAACAACTTTTAATTAATTGTTTGGAAGAACTACCAGCCAAAAATATAGTAATTAATATGTATATTAGAACAAAATTTTGGTGATTCGCAGGCCAGTTTAATATTTCCTTAAAGTCTATGGCGCCCGGCTGGGCATTAGTGAAGCTTTTTCTGTTATCTTGATGAAGATGGTTTATACAAGGAAAAACAAACAGTAATTTATGATTTGTCCTTTTCTCGCAGGGCATACCTGATCGAGTAACTACAGAGGTGTTTATAGCCATGTCAAAGGCCCTTAACTTTATTAACCCTGATGAACTTTCAATGCAGTGCATATTGATTGCTTTGAACCGATTCCTCCAGGTATATTGAATTACCCCAACTTGACTTTTAGAAAAAATCTTGTTCCTTTTCCTTCATATAATATTGGTATGGAGGAAAATAAAAGATACAATGTTATGGCTTCATGTGCACCATTAATGCAAGTTTAGAACCACGTCTTTTTCGTTTCCTTGTATATTACATGGAGCTTAAGTCAGATCTCAGAAGCAACATATCCTGAGTAGTATTCCTTGTGGAAGATCAGTCTCAATTTTGGATGGGTTCCTTCTCTGTATGCACTACTGTATATAGATGATCAGTAAACTATTACTTAGTGGACAAATTTTTGTTACAACCTAGCATTTGCTGATCAGCAGATTTGCACACATGTAGGAGAAACACGGTTCCAAGATGGCTTTCTTGGATGGTAGTCCCCCCGAGAGACTCTGTGCTCCAATTGTTGATCATATCCAGTCATTGGGCGGTGAAGTCCGAACTAATTCCCGAATACAGAAAATTGATCTAAATAACGATGGAACTGTGAAGAGTTTTGTACTAAATAATGGGAGCGTGATTGAAGCAGATGCGTATGTGTTCGCCACTCCAGGTTTGGCACAGTTTTTTTTGTTTAAGACCACTTAGTGGGAAAAGGCTTTGTTGTTGTTGTTGTTGGTCTTTAATTTATATTCAAGAGATTGTTTGATTTTCTTTTAAACAGTTGATATCCTAAAGCTTCTATTGCCTGAAAACTGGAAAGAGATGCCATATTTCAAGAAATTGGAGAAATTAGTTGGAGTTCCAGTTATCAATGTTCACATATGGTAA

Additional Table S1 Selection of target sites and off target possibility

| Target site | Target sequence | Position | Strand | Off target possibility |
| --- | --- | --- | --- | --- |
| Target site A | TCTCTCGCTTCTAGTAGTATAGG | 3 extron | antisense strand | 0.284 |
| Target site B | GCAGCATGGAAAGATAGTGATGG | 4 extron | sense strand | 0.46 |
| Target site C | ACAAGCCAGGGGAGTTCAGTCGG | 5 extron | sense strand | 0.198 |
| Target site D | TCAAACCGACTGAACTCCCCTGG | 5 extron | antisense strand | 0.308 |
| Target site E | ACCTCTGTAGTTACTCGATCAGG | 6 extron | antisense strand | 0.596 |

Red color marked bases are PAM sites


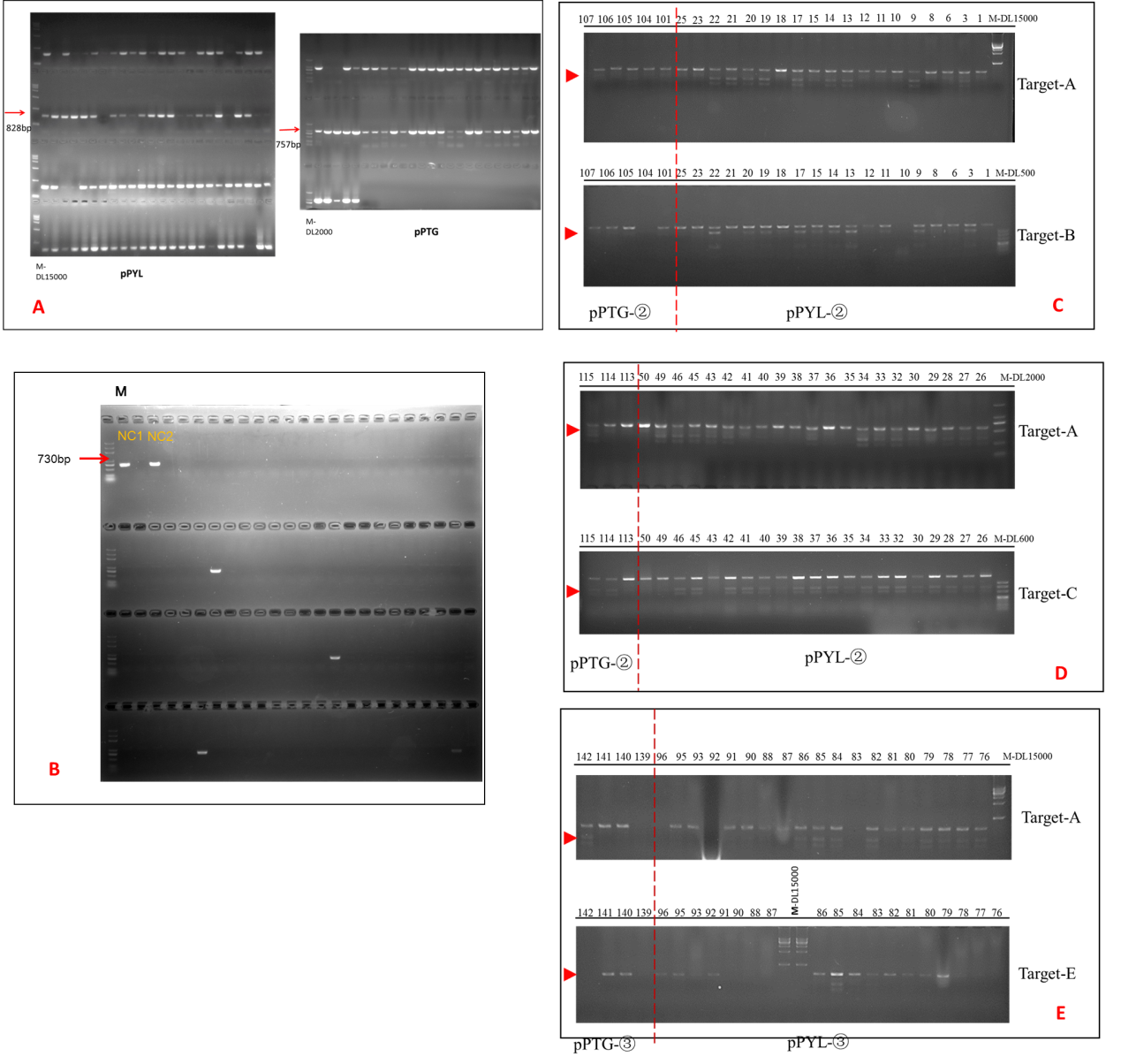


Additional Fig S1 Identification of T-DNA insertion and indel detection by T7E1 assay.

A: Transgenic positivity check in hygromycin- or Kan-resistant callus lines with Cas9-specific primers SP-L1/SP-R-L or SP-DL/SP-R-W. Left: Representation of CRISPR/Cas9 system. Right: Representation of PTG/Cas9 system. B: Transgenic positivity check in hygromycin- or Kan-resistant callus lines with *Agrobacterium*-specific primers VCF/VCR. Yellow text indicates *Agrobacterium* as PCR negative control. C-E: T7E1 assay to detect CRISPR-induced mutations in hygromycin- or Kan-resistant calli. Red arrowheads indicate the fragments digested by T7E1.
